# Variation in the morphology and effector profiles of *Exserohilum turcicum* isolates associated with the Northern Corn Leaf Blight of maize in Nigeria

**DOI:** 10.1186/s12870-023-04385-7

**Published:** 2023-08-10

**Authors:** Faith A. Bankole, Baffour Badu-Apraku, Abiodun O. Salami, Titilayo D.O. Falade, Ranajit Bandyopadhyay, Alejandro Ortega-Beltran

**Affiliations:** 1 International Institute of Tropical Agriculture, Ibadan, Nigeria; 2 First Technical University, Ibadan, Nigeria

**Keywords:** Disease management, Breeding for durable resistance, Pathogenicity, Virulence factors, Effectors

## Abstract

**Background:**

Maize production in lowland agro-ecologies in West and Central Africa is constrained by the fungus *Exserohilum turcicum*, causal agent of Northern Corn Leaf Blight (NCLB). Breeding for resistance to NCLB is considered the most effective management strategy. The strategy would be even more effective if there is adequate knowledge of the characteristics of *E. turcicum* in a target region. Maize leaves showing NCLB symptoms were collected during field surveys in three major maize growing areas in Nigeria: Ikenne, Ile-Ife, and Zaria during 2018/2019 and 2019/2020 growing seasons to characterize *E. turcicum* populations interacting with maize using morphological and molecular criteria.

**Results:**

A total of 217 *E. turcicum* isolates were recovered. Most of the isolates (47%) were recovered from the Ikenne samples while the least were obtained from Zaria. All isolates were morphologically characterized. A subset of 124 isolates was analyzed for virulence effector profiles using three primers: *SIX13-*like, *SIX5-*like, and *Ecp6*. Inter- and intra-location variations among isolates was found in sporulation, growth patterns, and presence of the effectors. Candidate effector genes that condition pathogenicity and virulence in *E. turcicum* were found but not all isolates expressed the three effectors.

**Conclusion:**

Morphological and genetic variation among *E. turcicum* isolates was found within and across locations. The variability observed suggests that breeding for resistance to NCLB in Nigeria requires selection for quantitative resistance to sustain the breeding efforts.

## Background

Maize (*Zea mays* L.) is a source of carbohydrates, vitamins, minerals, and proteins. Over 4.5 billion people in developing countries obtain ~ 30% of their calories from maize. In sub-Saharan Africa (SSA), maize is the most widely grown and consumed staple food with an estimated 50% of the populace depending on maize as the primary staple [1, 2]. Maize ranks first in the world cereal production (1.15 billion tons) with the United States of America as the largest producer (~ 50% of the total production) [3]. In Africa, South Africa is the highest maize producer (11.3 m tons), followed by Nigeria (11.0 m tons), and Ethiopia (9.6 m tons) [3].

Production and productivity of maize in SSA are confronted with myriads of biotic and abiotic stresses [4]. These include low soil fertility; drought, heat stresses; combined heat and drought stresses; parasitic weeds [*Striga hermonthica* (Del.) Benth]; insect pests; and several plant pathogenic fungi, bacteria, viruses, and nematodes [1, 5, 6]. Globally, fungi are prominent causal agents of plant diseases and pose a major constraint to maize production [7, 8]. Foliar diseases by fungi can be destructive, and one of the most damaging is the Northern Corn Leaf Blight (NCLB) incited by *Exserohilum turcicum* (Passerini) Leonard and Suggs. The disease is prevalent in highland regions [9]. However, the spread to lowlands has been documented [4, 10].

The fungus *E. turcicum*, a hemi-biotrophic ascomycete [11], survives in humid conditions and moderately low (17–28 °C) temperatures [12]. As a biotroph, the fungus penetrates the host either through suppression or evasion of the host defense structures while maintaining the viability of the host [13, 14]. This is immediately followed by the necrotrophic phase, characterized by increased production of cell wall-degrading enzymes (CWDEs), secondary metabolites, and fungal biomass leading to necrosis [15, 16]. CWDEs aid penetration and aggressive colonization of host cells [17]. Fungal effectors are required for penetration and subsequent host colonization [18, 19]. Lo Presti et al. and Jones and Dangl reported that fungal pathogens produce effectors that modulate host defense structures and physiology to penetrate, evade detection, and acquire nutrients [18, 20]. However, an immune response can be elicited if the corresponding resistance gene (R-gene) of the host is recognized by the corresponding effector gene [21].

The photosynthetic potential of maize is significantly reduced by *E. turcicum* infection and this invariably affects grain yield [22]. The premature death of infected plants has been reported when the disease epidemic begins at an early stage [11, 23]. This is consequent upon the alteration of the physiological activities of the infected host [24]. Additionally, increases in temperature and rate of transpiration have been associated with infected plant parts as the severity increases. The progress of infection from the lower leaves to the leaves above the ear before tasseling or silking becomes very severe, especially, when the environment favors the establishment of the pathogen [11, 25].

NCLB can be managed through cultural methods (e.g., crop rotation with non-host crops or complete burying of disease crop residues through tillage operations), chemical control, and host plant resistance (HPR) [6, 26]. HPR is an economically feasible, safe, durable, sustainable, and eco-friendly control method for managing plant diseases [27, 28]. However, understanding variations among and within populations of causal agents of a disease is important for developing appropriate breeding strategies [29]. Diversity within communities of plant pathogenic fungi is shaped by sexual reproduction, clonality, and/or mutation. A mixed system of reproduction, i.e., sexual and clonal, has been reported in *E. turcicum* populations. Although the sexual stage of *E. turcicum* is relatively uncommon in nature, substantial evidence of recombination in tropical populations exists [30–32].

Genetic diversity in *E. turcicum* has been studied via different methods [30–34]. Haasbroek et al. developed 13 microsatellite markers to study the genetic variability of *E. turcicum* in South Africa and were able to distinguish isolates with specificity to either maize or sorghum [35]. In addition, identification of genes facilitating host colonization during the biotrophic and necrotrophic stages has been effective for the classification of different strains [16]. Also, *formae speciales* of *Setosphaeria turcica* in China were identified and their genetic diversity studied using ‘universally primed polymerase chain reaction (PCR)’ [36]. Human et al. employed a time-course RNA sequencing method and identified several enzymes, secondary metabolites, effectors, and candidate genes that moderate the pathogenicity of *E. turcicum* [16]. The effector gene *Ecp6*, has been characterized for its role in pathogenicity [3, 16]. In addition, secreted in xylem (*SIX*) genes (*SIX5-*like and *SIX13*-like) modulate host responses and can interact with the R-genes when recognized by the host [37]. The expressions of *Ecp6* and *SIX13-*like genes are associated with the establishment of the pathogen during the biotrophic stage [16] while *SIX5-*like genes facilitate the passage of the *Avr2* effector genes to adjacent cells [38].

Adequate knowledge of both the genetic basis of resistance and diversity within communities of the causal agents of the disease are of prime importance in breeding for HPR. However, in Nigeria, although significant work to elucidate the genetic basis of the resistance in early-maturing and extra-early-maturing maize germplasm has been done [10, 23, 39], there is a lacuna in information regarding the genetic diversity among *E. turcicum* populations interacting with maize. Therefore, the present study investigated variability among *E. turcicum* populations associated with maize grown in three agro-ecologies of Nigeria during two seasons. In addition to morphological characterization, a subset of isolates of *E. turcicum* were subjected to molecular assays to determine the presence or absence of plant disease effectors. The results could contribute to refined breeding efforts for the development of resistance in maize to various pathotypes of *E. turcicum* interacting with maize in Nigeria.

## Results

### Morphological characterization of *Exserohilum turcicum* isolates

**Fig. 1:**
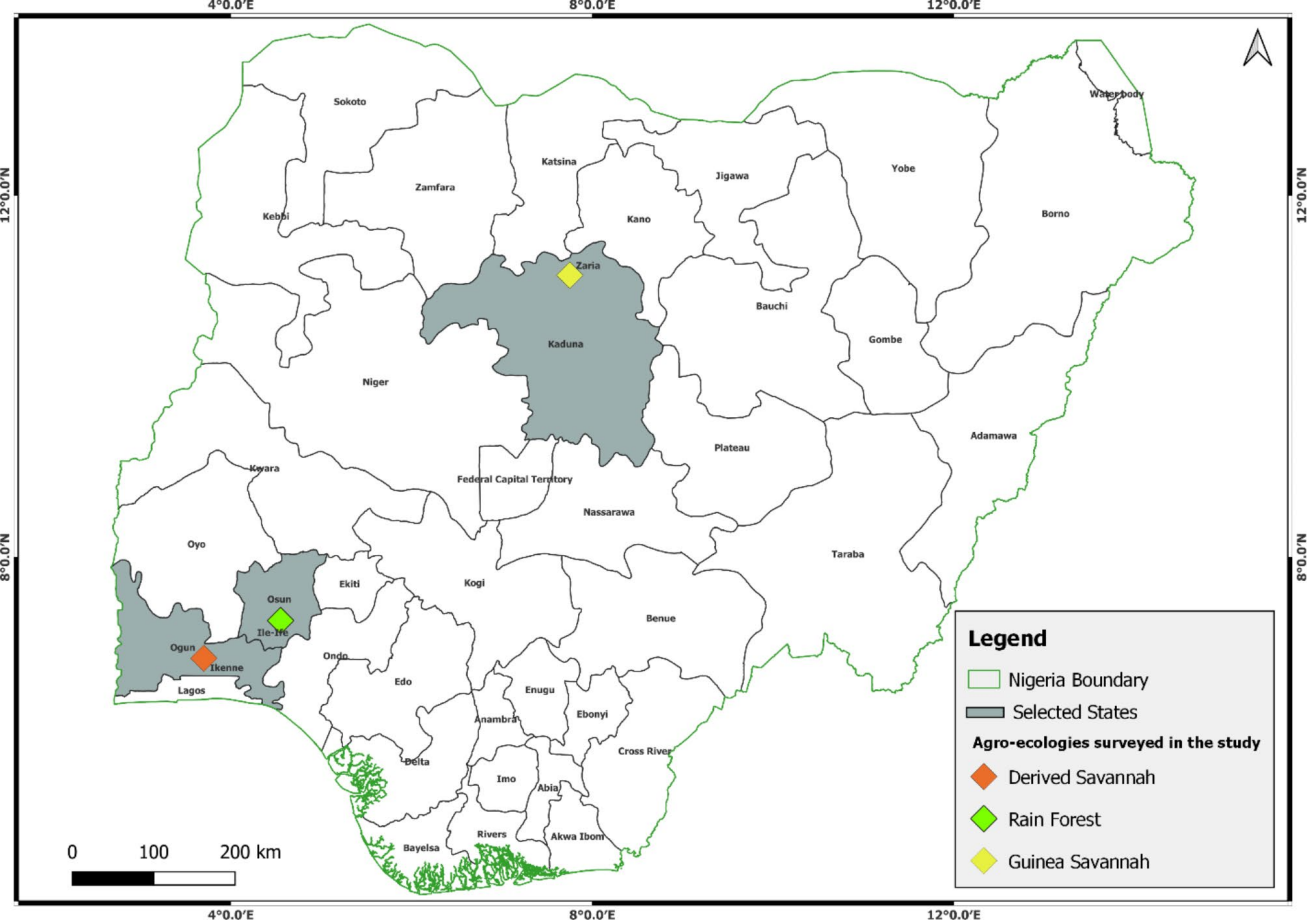
Map of Nigeria showing locations where leaves of maize plants showing northern corn leaf blight were collected

**Fig. 2:**
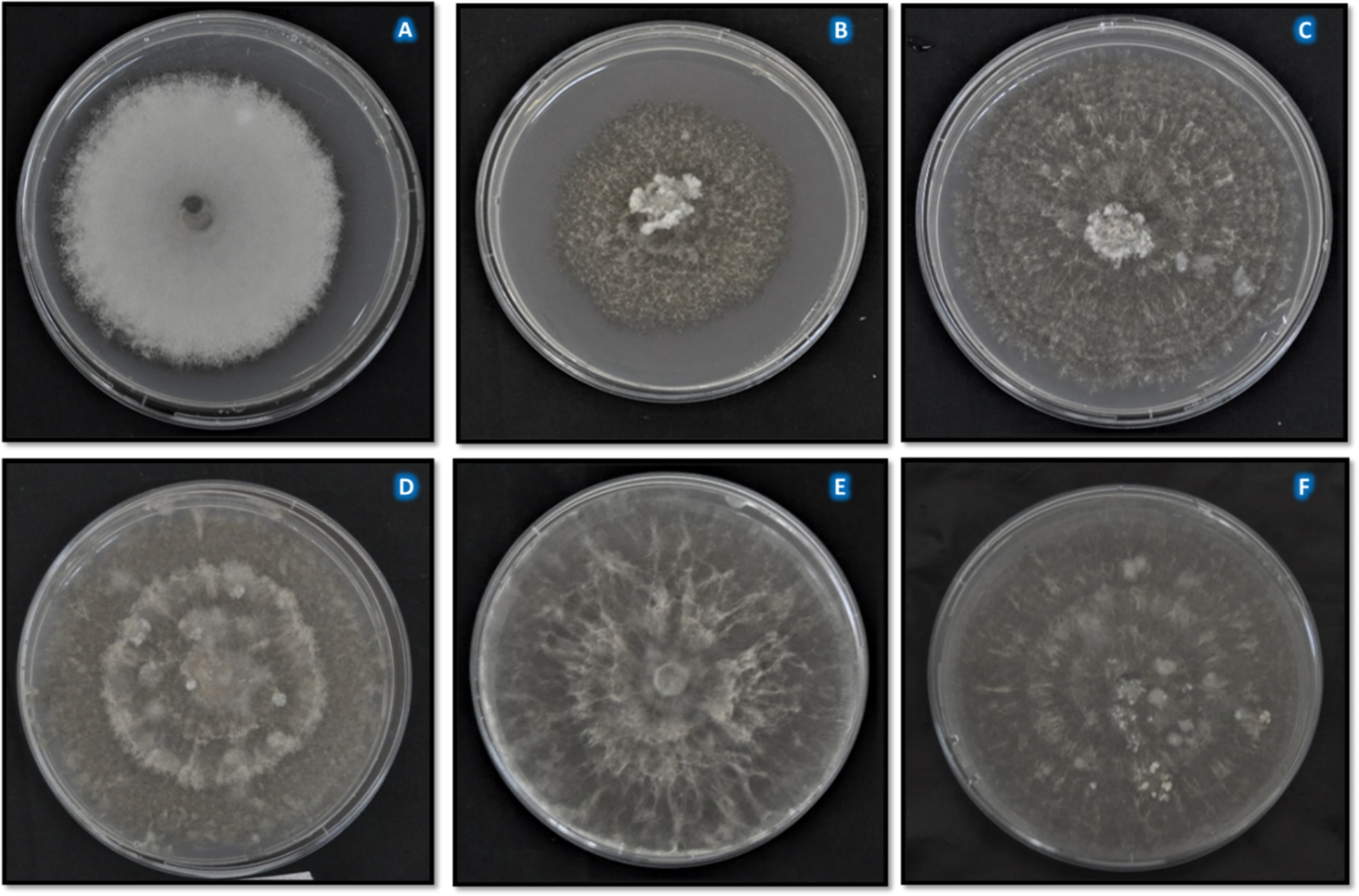
Mycelia growth of *Exserohilum turcicum* isolates on potato dextrose agar. ETDS 5 (**A**) and ETDS 46 were isolated from Ile-Ife (**B**), ET-AB-1 A-2 (**C**) and ET-AB-1 A-3 (**D**) are Zaria isolates, while 10-IK-20G (**E**) and ET-IK-7-7B-3 (**F**), were isolated from Ikenne

A total of 217 isolates of *E. turcicum* were recovered from Ikenne (102), Ile-Ife (61), and Zaria (54) (Fig. 1). Morphological variations based on color, radial mycelia growth (RMG), and growth rate were observed among and within locations (Fig. 2). Isolates grew radially on PDA with black fluffy but irregularly interspersed with whitish mycelia and aerial hyphae in some isolates (e.g., ETDS 5 from Ile-Ife; Fig. 2). Generally, the isolates produced black pigmentation on potato dextrose agar (PDA) (Fig. 2).

**Fig. 3:**
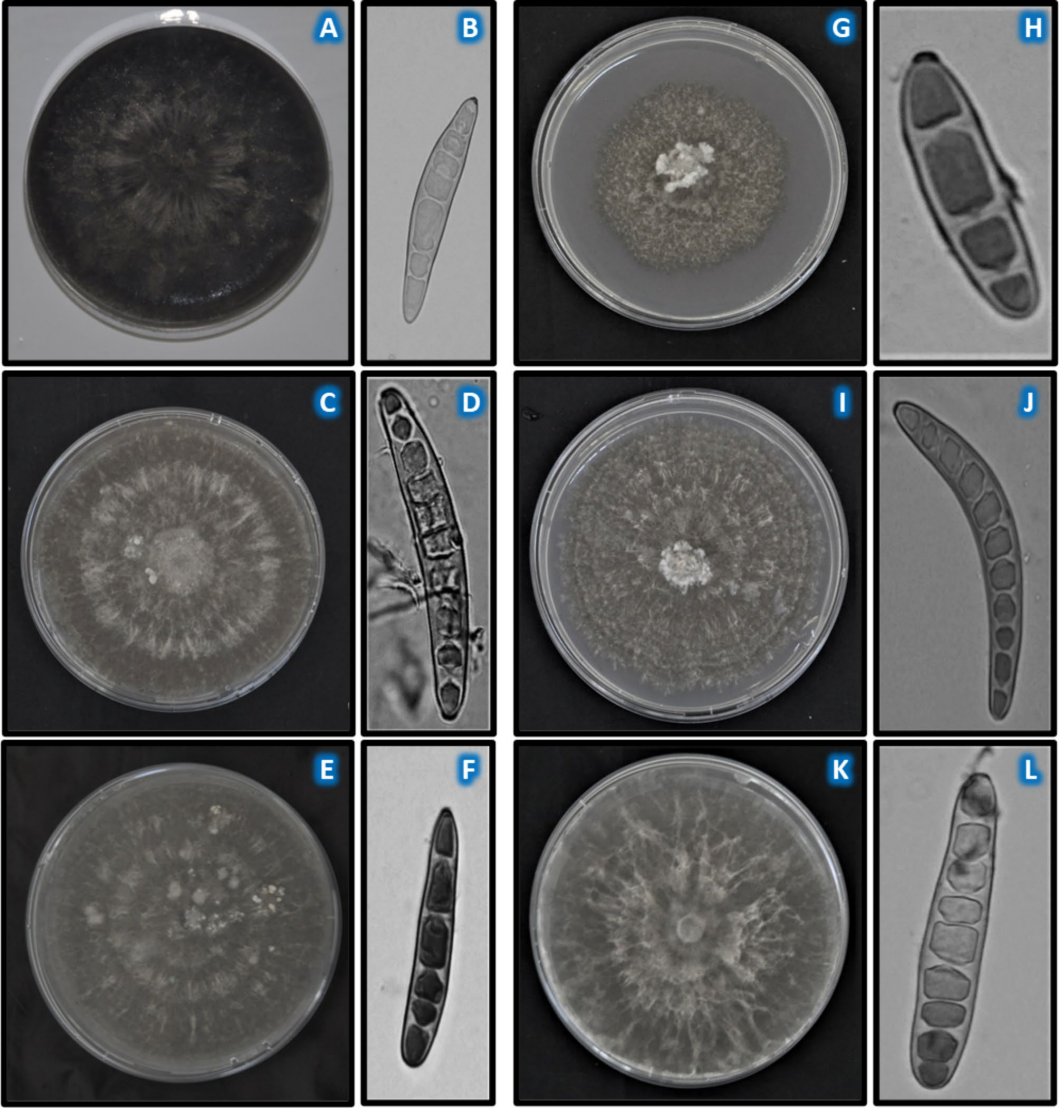
Morphology of *Exserohilum turcicum* isolates grown on PDA. Colony (**A**) and spore (**B**) of NGETIB16-13; colony (**C**) and spore (**D**) of NGETIK16-6; colony (**E**) and spore(**F**) of ET-IK-7-7B-3; colony (**G**) and spore (**H**) of ETDS 46; colony (**I**) and spore (**J**) of ET-AB-1A2; colony (**K**) and spore (**L**) of 10-IK-20G. All spores magnified at ×40

Generally, the conidia were cylindrical, tapering from the middle with transverse septations and conspicuously protruded hilum (Fig. 3). The conidiophores were cylindrical, septate, olivaceous brown, and bear single conidia. The conidia varied in the number of transverse septations, sporulation, and size (Fig. 3). The number of septation of the conidia in Ikenne isolates, ranged from 2 to 10 (Table 1), while in Ile-Ife and Zaria isolates it ranged from 2 to 4 (Tables 2 and 3). The maximum number of transverse septations across locations ranged from 8 to 13. The highest number of septation (13) was recorded among Ikenne isolates.

**Table 1:** Morphological characteristics of *Exserohilum turcicum* isolates from Ikenne in 2018/2019

Isolate code	Number of septa		Spore length (µm, X ± SE_0.05_)	Spore count (X ± SE_0.05_)	SPFM	RMG 14DAP
	Min.	Max.				
NGETIK-16-8	3	12	5.5 ± 0.6	18 ± 0.2	Good	24.0
NGETIK-16-11	2	9	5.2 ± 0.3	23 ± 0.3	Very Good	31.0
IK-5-1 A-4	2	11	5.9 ± 0.2	25 ± 0.1	Excellent	40.0
NGET1B16-13	3	13	5.4 ± 0.1	18 ± 0.1	Good	49.0
10-IK-18 C	10	13	5.2 ± 0.3	19 ± 0.1	Good	54.7
ET-IK-21	3	11	5.9 ± 0.4	24 ± 0.2	Very Good	62.4
10-IK-20G	9	13	4.8 ± 0.4	20 ± 0.3	Very Good	65.4
IK-7-7B-3	3	11	8.0 ± 0.4	24 ± 0.1	Very Good	68.7
ET-IK-24	2	8	4.8 ± 0.3	21 ± 0.2	Very Good	72.3
**LSD**_**0.05**_						**16.8**

Min: minimum. Max: maximum, SE: standard error, RMG: radial mycelia growth (cm^2^); DAP: days after plating, LSD: least significant difference, X: mean values, spores per microscopic field (SPMF) as excellent: ≥ 25 SPMF; very good: 20 to 24 SPMF; good: 15 to 19 SPMF; fair: 10 to 14 SPMF; and poor: 1 to 9 SPMF at ×25 magnification

**Table 2:** Morphological characteristics of selected *Exserohilum turcicum* isolates from Ile-Ife in 2018

Isolate code	Number of septa		Spore length (µm, X ± SE_0.05_)	Spore count (X ± SE_0.05_)	SPFM	RMG 14DAP
	Min.	Max.				
ETDS 8	2	10	5.2 ± 0.4	19 ± 0.2	Good	20.4
ETDS 134	2	8	3.2 ± 0.3	4 ± 0.2	Poor	21.7
ETDS 5	4	11	5.3 ± 0.5	19 ± 0.2	Good	22.5
ETDS 46	4	11	7.3 ± 0.3	29 ± 0.2	Excellent	40
ETDS 122	2	11	7.1 ± 0.5	18 ± 0.2	Good	45.1
ETDS 108	3	10	4.8 ± 0.8	10 ± 0.2	Fair	53.8
ETDS 52	3	12	6.9 ± 0.6	20 ± 0.2	Very Good	55.5
ETDS 126	2	10	5.2 ± 0.4	21 ± 0.2	Very Good	56.6
ETDS 140	3	11	9.6 ± 1.0	21 ± 0.2	Very Good	62.4
ETDS 92	2	9	5.6 ± 0.3	25 ± 0.2	Excellent	62.8
ETDS 111	2	9	5.0 ± 0.4	19 ± 0.2	Good	64
ETDS 149	3	10	6.3 ± 0.4	20 ± 0.2	Very Good	65.6
ETDS 93	4	9	5.3 ± 0.4	21 ± 0.2	Very Good	69.3
ETDS 97	2	9	3.9 ± 0.8	9 ± 0.2	Poor	71.8
**LSD**_**0.05**_						**15.8**

Min: minimum. Max: maximum, SE: standard error, RMG: radial mycelia growth (cm^2^); DAP: days after plating, LSD: least significant difference, X: mean values, spores per microscopic field (SPMF) as excellent: ≥ 25 SPMF; very good: 20 to 24 SPMF; good: 15 to 19 SPMF; fair: 10 to 14 SPMF; and poor: 1 to 9 SPMF at ×25 magnification

**Table 3:** Morphological characteristics of selected *Exserohilum turcicum* isolates recovered from Zaria in 2019

Isolate code	Number of septa		Spore length (µm, X ± SE_0.05_)	Spore count (X ± SE_0.05_)	SPFM	RMG 14DAP
	Min.	Max.				
AB19-11A1	2	9	5.3 ± 0.4	18 ± 0.1	Good	28.4
AB19-13A4	2	10	5.4 ± 0.1	19 ± 0.3	Good	34.6
AB19-19A2	2	9	4.2 ± 0.4	17 ± 0.3	Good	39.3
AB19-10A1	2	9	4.2 ± 0.4	19 ± 0.2	Good	40.3
AB19-12B1	2	9	5.2 ± 0.4	17 ± 0.1	Good	42.0
AB19-11B2	2	9	5.0 ± 0.4	19 ± 0.4	Good	49.4
AB19-20B1	2	11	3.6 ± 0.1	18 ± 0.3	Good	50.1
AB19-8B1	2	10	5.4 ± 0.4	20 ± 0.2	Very Good	51.8
AB19-21A1	2	8	3.0 ± 0.4	19 ± 0.2	Good	56.1
AB19-1A3	3	10	5.2 ± 0.4	21 ± 0.1	Very Good	59.3
AB19-3 A-1	2	10	6.6 ± 0.4	20 ± 0.3	Very Good	59.3
AB19-4 A-1	4	9	4.9 ± 0.4	19 ± 0.2	Good	62.8
**LSD**_**0.05**_						**14.9**

Min: minimum. Max: maximum, SE: standard error, RMG: radial mycelia growth (cm^2^); DAP: days after plating, LSD: least significant difference, X: mean values, spores per microscopic field (SPMF) as excellent: ≥ 25 SPMF; very good: 20 to 24 SPMF; good: 15 to 19 SPMF; fair: 10 to 14 SPMF; and poor: 1 to 9 SPMF at ×25 magnification

**Fig. 4:**
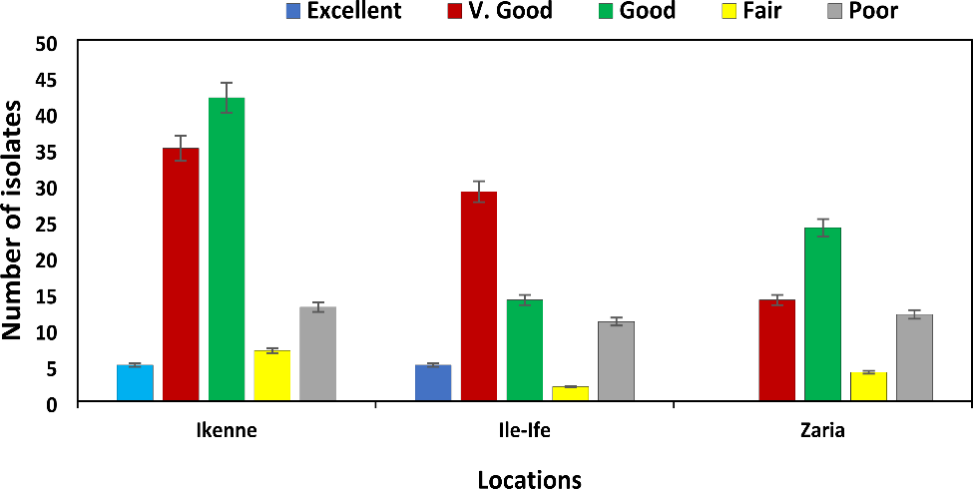
Classification of *Exserohilum turcicum* isolates recovered from Ikenne, Ile-Ife and Zaria in 2018/2019 based on spore count per microscopic field. Excellent: ≥ 25 spores per microscopic field at ×25 (SPMF); very good: 20 to 24 SPMF; good: 15 to 19 SPMF; fair: 10 to 14 SPMF; and poor: 1 to 9 SPMF (Harlapur et al., 2007)

The number of spores per microscopic field (SPMF) is presented in Fig. 4. Isolates from Ikenne (102) had representatives in each category. However, only five had excellent SPMF. Thirty-five isolates had very good SPMF, 42 had good SPMF, and only seven and 13 isolates had fair and poor SPMF, respectively (Fig. 4). Similar distribution in terms of sporulation was observed for Ile-Ife isolates. Although none of the Zaria isolates had excellent sporulation, the other classes were observed (Fig. 4).

Significant differences were observed for spore length and RMG of the isolates from each location. The conidia length and the RMG of Ikenne isolates is presented in Table 1. IK-7-7B-3 had the largest spore length while 10-IK-20G had the shortest (Table 1). The RMG of Ikenne isolates 14 days after plating (DAP) ranged from 24.0 cm^2^ for NGETIK-16-8 to 72.3 cm^2^ for ET-IK-24. Similar results were observed for Ile-Ife isolates. There were significant differences among isolates for spore length and RMG at 14 DAP (Table 2). ETDS 134 had the shortest conidia length while ETDS 140 had the largest (Table 2). In addition, the RMG at 14 DAP ranged from 20.4 cm^2^ for ETDS 8 to 71.8 cm^2^ for ETDS 97. The morphological characteristics of Zaria isolates are presented in Table 3. The spore length of AB19-21A1 was 3.0 μm and was significantly shorter than AB19-3A1 which had the largest spore. AB19-11A1 had the lowest RMG at 14 DAP while AB19-4-A1 had the largest (Table 3). The correlation analysis showed no significant relationship between the spore count and the RMG for all the isolates across locations, however, positive (*r* = 0.41, *P* < 0.05) significant relationships were observed for spore count and spore length as well as for septation and spore length (*r* = 0.84, *P* < 0.05) (Table 4). For example, IK-5-1 A-4 (from Ikenne) and ETDS 46 (from Ile-Ife) had excellent sporulation but poor RMG, while ET-IK-24 had very good sporulation and the highest RMG. ETDS 97 had poor sporulation and the highest RMG among Ile-Ife isolates.

**Table 4:** Pearson correlation coefficient of radial mycelia growth, spore count, spore length and septation of *Exserohilum turcicum* isolates from Ikenne, Ile-Ife and Zaria in 2018/2019

	RMG	Spore length	Spore count	Septation
**RMG**	1	0.10	0.01	0.07
**Spore length**		1	0.41**	0.84**
**Spore count**			1	0.21
**Septation**				1

** significance at *P < 0.01*; RMG: radial mycelia growth

**Fig. 5:**
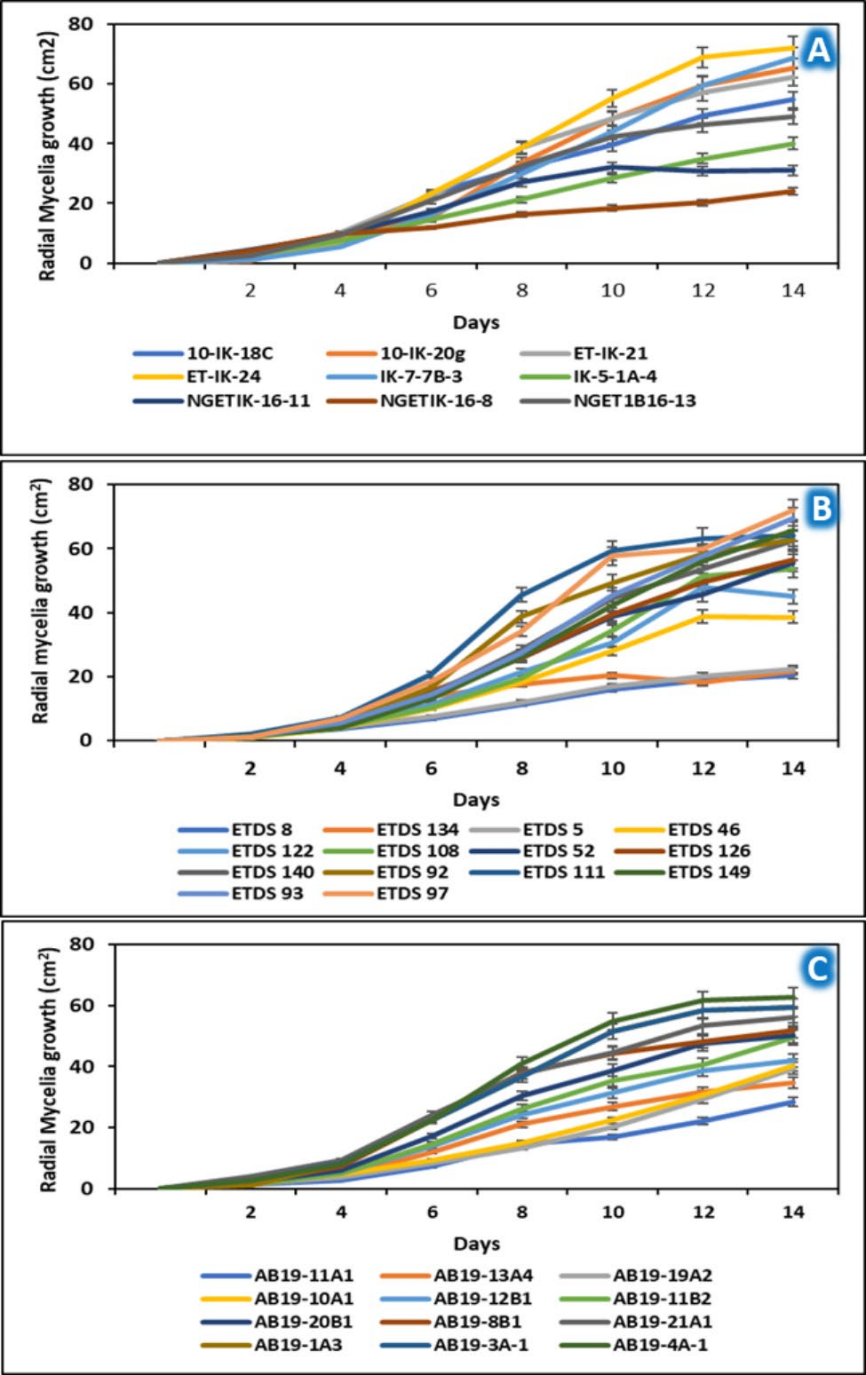
Growth rates of selected *Exserohilum turcicum* isolates recovered from Ikenne (**A**), Ile-Ife (**B**), and Zaria (**C**), during 2018/2019 growing seasons

The growth rate of selected *E. turcicum* isolates is presented in Fig. 5. For Ikenne isolates, NGETIK-16-8 had the least growth rate, followed by NGETIK-16-11. However, the highest growth rate was recorded for ET-IK-24. Similarly, significant (*P* < 0.05) differences were found for growth rates of Ile-Ife isolates. For example, the least growth rate was recorded for ETDS 8 while ETDS 97 had the highest, a poor sporulating isolate. Furthermore, the Zaria isolates also had significant variations in relation to growth rate. AB19-4 A-1, classified as a good sporulating isolate, had the fastest growth compared to other isolates.

### Molecular characterization of a subset of *Exserohilum turcicum* isolates

**Fig. 6:**
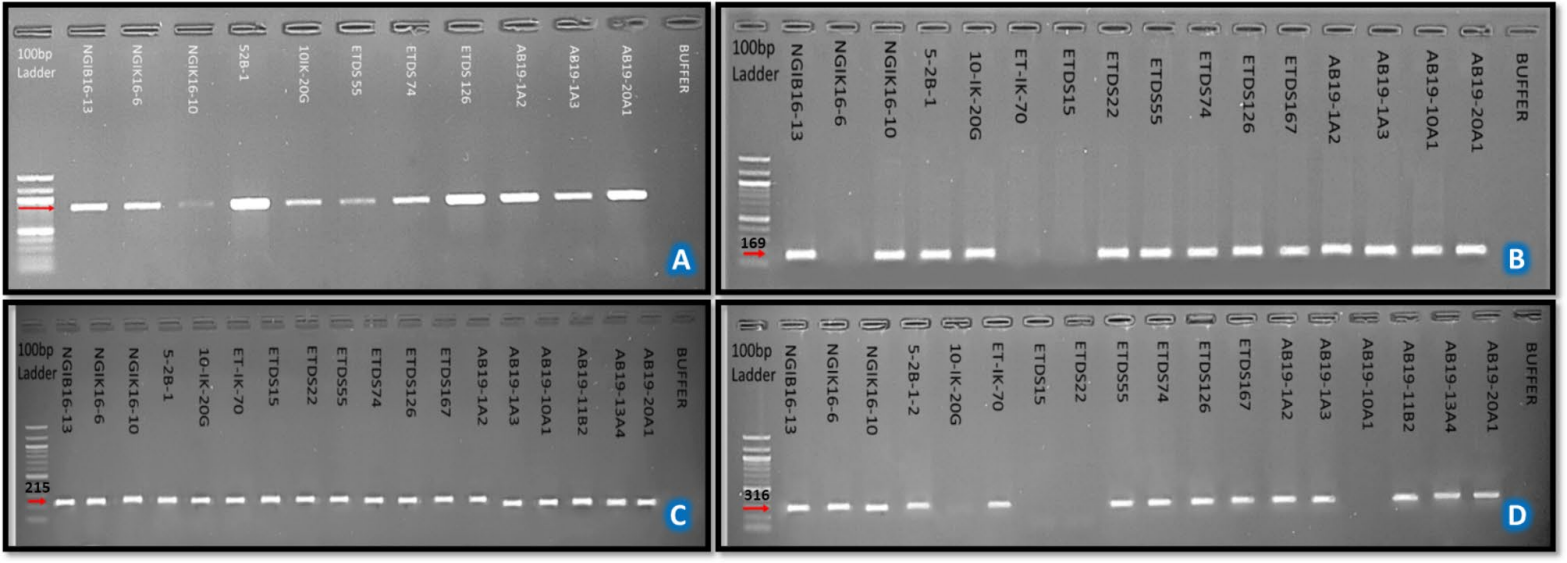
Gel electrophoresis picture showing the PCR results of selected *Exserohilum turcicum* isolates using the different primer pairs. **A**: Elongation factor *EF1*-α, **B**: *SIX13*-like effector gene, **C**: *SIX5*-like effector gene, **D**: *Ecp6* effector gene

**Fig. 7:**
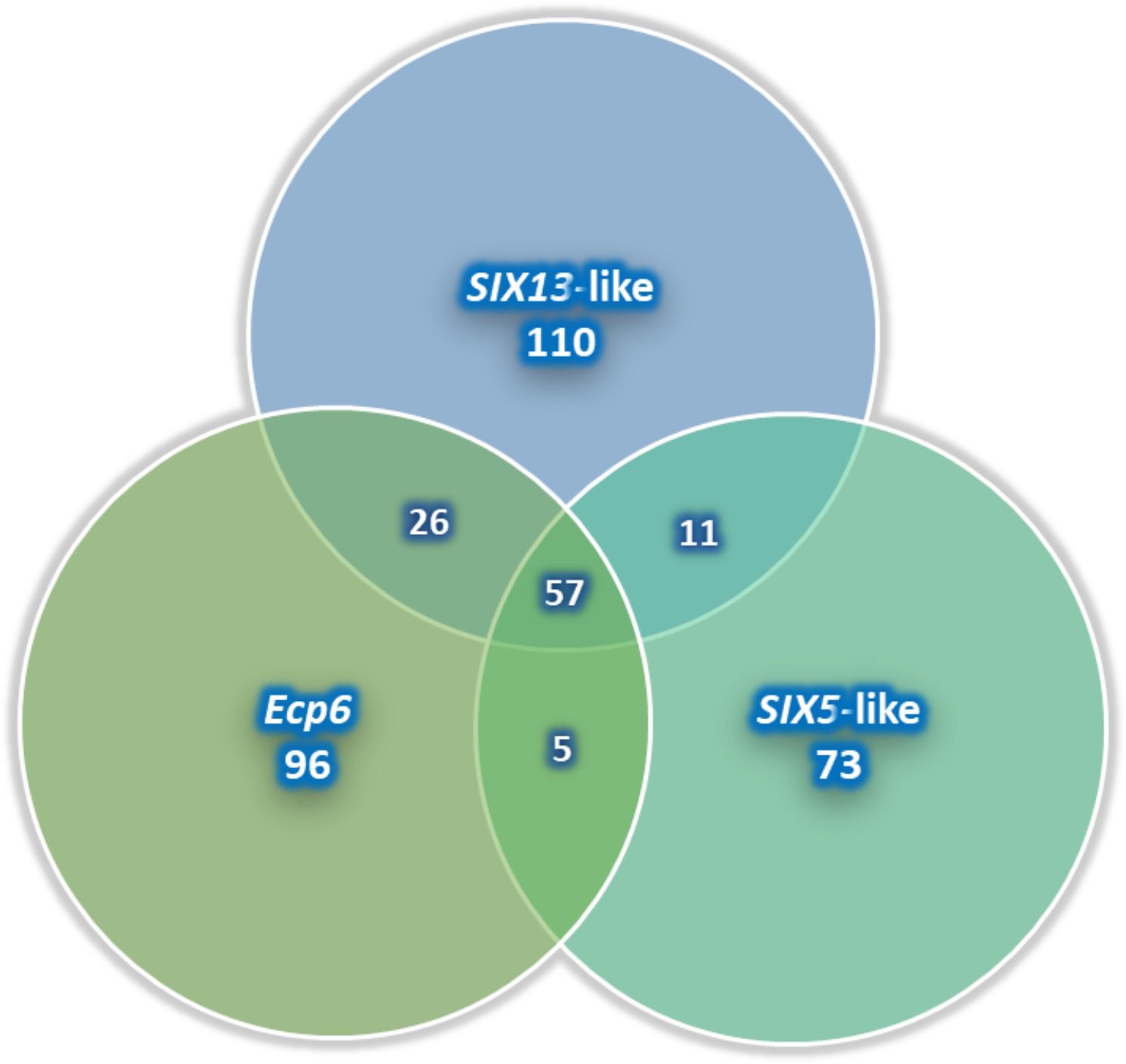
Venn diagram showing the amplification patterns of segments of three effector genes by *Exserohilum turcicum* isolates collected across the three areas in Nigeria

Of the *E. turcicum* isolates (217) identified and characterized morphologically, only 56, 47, and 21 from Ikenne, Ile-Ife, and Zaria, respectively, were characterized molecularly. The isolates were selected for representative groups based on morphological features such as RMG, sporulation, conidia size, and mycelial pattern. All selected isolates amplified the reference *EF1-α* primer pair (Fig. 6a). On the other hand, amplification of segments of the three *E. turcicum*-specific primer pairs for effector genes varied. Overall, 89% of the isolates amplified the *SIX13*-like primer pair: 43 of the 56 Ikenne isolates amplified the *SIX13*-like primer pair; all except one of the 47 Ile-Ife isolates amplified it while all Zaria isolates amplified it (Fig. 6b). There were notable variations for the *SIX5*-like amplification compared to *SIX13*-like. Overall, 59% of the isolates amplified the *SIX5*-like primers: 27/29 Ikenne isolates, 27/47 Ile-Ife isolates, and 19/21 Zaria isolates (Fig. 6c). For *Ecp6* primer pair there were amplifications for 42/56 Ikenne isolates, 34/47 Ile-Ife isolates, and 20/21 Zaria isolates. Overall, 77% of the isolates amplified the *Ecp6* primer. *Ecp6* amplification bands of selected isolates is presented in Fig. 6d. Of the 124 isolates characterized, 57 amplified the three effectors. Most of the Zaria isolates amplified the three effectors. A Venn diagram showing number of isolates amplifying 1, 2, or 3 effectors is presented in Fig. 7.

## Discussion

The present study is the first survey of *E. turcicum* associated with maize in different agro-ecologies in Nigeria. The study was conducted to determine whether morphological and molecular variability among isolates interacting with maize in Nigeria exists. A total of 217 isolates were recovered in three locations: Ikenne (102), Ile-Ife (61), and Zaria (54). The results of the characterization of the isolates using morphological and molecular criteria revealed variations within and among the locations. The relative humidity and annual rainfall of the study locations are higher in Ikenne compared to Zaria [10]. These climatic conditions might have contributed to the higher incidence of the disease and, hence the numerous isolates of *E. turcicum* recovered from Ikenne compared to Ile-Ife and Zaria.

The recovered isolates varied significantly in morphology including colony color, growth pattern, conidia structures, size, and RMG. Several colony colors of *E. turcicum* have been reported on PDA: from white to green-white, olivaceous gray to brown with black pigmentation [10, 40, 41]. The range of shades of colony morphology colors observed in the present study were similar to those previously reported. The conidia of all isolates had a conspicuous protruded hilum, as expected [12]. The number of septation and conidia length conformed with those of previous reports [41]. Some isolates had a high spore reproduction rate, which could be used as a proxy for survival and spread of plant pathogens [42]. Different categories of SPMF following a modified scale [43] were recorded. Most isolates across locations had > 15 SPMF indicating their rapid spore production under laboratory conditions. Badu-Apraku et al. recorded high incidence and severity of NCLB in the study locations which could be linked to the rapid growth and multiplication of *E. turcicum* in the study area [10]. Generally, pathogens spread by rapid production and dissemination of spores [43]. Therefore, the different growth rates and SPMF recorded in the present study suggest that the pathogen has the potential to rapidly spread and this could be a challenge to NCLB management in the study areas. The observed morphological variation of *E. turcicum* could be a result of varying environmental conditions of the study area and the survival strategy of the pathogen. Anwer et al. [41] and Abebe and Singburaudom [44] reported that environmental conditions could result in morphological variations in *E. turcicum* populations.

In the present study, there was variability among and within locations in isolate expression of effector genes conditioning pathogenicity and virulence. The choice of the method and the target genes adopted was based on the study of Human et al. [16]. They identified several enzymes, secondary metabolites, effectors, and candidate genes moderating *E. turcicum* pathogenicity and reported that the expression of *Ecp6*, *SIX13-*like, and *SIX5-*like effector genes were associated with the establishment of the pathogen during the biotrophic stage. Xue et al. [45], Niu et al. [37], and Cao et al. [38] reported similar results. However, in the present study, the expression of effector genes differed among the isolates. The number of isolates expressing only one of the following three effector genes are in parenthesis: *SIX13-*like (13), *SIX5-*like (1), and *Ecp6* (7). There were 28 isolates producing both *SIX13-*like and *Ecp6* effector genes, 11 producing both *SIX13-*like and *SIX5-*like, and five producing both *Ecp6* and *SIX5-*like (Fig. 7). Isolates from the current study that produced the three effector genes are more virulent compared to the others. In our previous studies, *E. turcicum* isolate NGETIB16-13, which amplified the three effector genes, was the most virulent isolate and therefore was used in resistance screening studies in the field [10, 23, 28, 39].

Several studies have reported the genetic relatedness of *E. turcicum* isolates from different locations. For example, McDonald and Linde reported a high potential of gene flow among *E. turcicum* isolates which portend genetic variations due to sexual reproduction [46]. Similarly, Borchardt et al. reported high genetic diversity, weak linkage disequilibrium and near-equal mating-type genes ratio for *E. turcicum* haplotypes, which provided evidence for frequent sexual recombination in populations from tropical regions of Kenya, Mexico, and China [47]. However, Human et al. provided evidence for clonal lineages of *E. turcicum* races among isolates recovered from locations that were separated by > 700 km in South Africa [16]. It is unclear which phenomenon conditions sexual reproduction in *E. turcicum*. The varying morphological and effector profiles found in the current study across and within locations suggest that the examined populations are not clonal. Therefore, further research is needed to determine the effects of mutation, marker loss, parasexuality, and/or recombination on the observed variations in effector production.

## Conclusion

This is the first report on *E. turcicum* associated with maize fields in different agro-ecologies in Nigeria. From three test locations —Ikenne, Ile-Ife and Zaria—217 isolates of *E. turcicum* were recovered and characterized using morphological and molecular traits. Morphological and genetic variation among *E. turcicum* isolates were found within and across locations. Candidate effector genes that condition pathogenicity and virulence in *E. turcicum* were found but not all isolates possessed the three genes, *SIX5-*like, *SIX13-*like, and *Ecp6*. The variations observed among the different isolates of *E. turcicum* evaluated in the present study calls for the review of the breeding methods for developing NCLB-resistant maize genotypes. These include the development of maize genotypes with quantitative resistance or multi-lines with specific resistant genes to different isolates of the pathogen. Relying on qualitative or monogenic resistance would not be enough for the management of the disease because it could easily be overcome by new strains of the pathogen.

## Methodology

### Sample collection and fungal isolation

Maize leaves with NCLB symptoms were collected from three test locations within contrasting agro-ecologies in Nigeria: Ikenne (6°53′ N, 3°42′ E), Ile-Ife (7°18̕′ N, 4°33̕′ E), and Zaria (11°7̕′ N, 7°45̕′ E), (Fig. 1). Using a purposive sampling method, leaves were collected, kept in labelled sample bags, and transferred to the laboratory for isolation of the causal agent. Five maize fields per location were sampled. Each field was divided into five plots and five leaf samples were obtained per plot. One to three sections per leaf showing NCLB symptoms were used for fungal isolations. Diseased tissues were surface sterilized for 30 s in 0.5% NaOCl and rinsed twice in sterile distilled water. The excised NCLB lesions were placed on sterile, moist filter paper in a Petri dish and incubated for 4 to 7 d at 25 °C to induce sporulation. Spores from each lesion were axenically transferred to potato dextrose agar (PDA), and incubated for 10 to 15 d. To obtain single spore cultures of each isolate, a plug of the sporulating fungal mycelia (14-d-old) was placed in a 40-ml vial containing 10 ml sterile distilled water. The mixture was briefly vortexed, serially diluted, and 10 µl of a 10^− 5^ suspension was spread using a sterile plastic spreader on water agar and incubated for 24 h. Spore germination was then examined with the aid of a dissecting microscope. A single germinating spore was isolated using a sterile pin, placed on PDA, and incubated at room temperature (25 °C) in the laboratory for 14 d.

### Morphological characteristics of *Exserohilum turcicum*

The characteristics (diameter, shape, size, and spore septation) of the spores of *E. turcicum* were determined by staining microscope mounts with lactophenol cotton blue stain and using a compound Olympus BX51 microscope at ×25 magnification. After taking the measurements with the calibrated ocular micrometer, photomicrographs of the spores were taken.

The radial mycelia growth (RMG) was calculated according to Harlapur et al. [43], with minor modifications. Briefly, plugs of the pure culture of each isolate were plated on PDA calibrated with a plastic ruler. Vertical and horizontal length growths (cm) of the mycelia of each isolate were measured for 14 d (at two days intervals). The RMG was calculated by multiplying the vertical and horizontal lengths with ∏ = 3.1416, assuming the shape of the Petri dish. The RMG and average spore length was subjected to analysis of variance (ANOVA) and isolates with rapid mycelial growth were identified. Production of spores by isolates was classified based on the number of spores per microscopic field (SPMF) as excellent: ≥ 25 SPMF; very good: 20 to 24 SPMF; good: 15 to 19 SPMF; fair: 10 to 14 SPMF; and poor: 1 to 9 SPMF at ×25 magnification [43]. The mean ± standard error (SE) for spore count and spore length were calculated while the relationships between spore length, spore count, and RMG were determined using Pearson correlation analysis. Mean ± SE, ANOVA, and correlation analysis were estimated using SAS v.9.1 (SAS Institute, Cary, NC, USA).

### Genomic DNA extraction

The methods of Sambrook et al. [48] and Callicott and Cotty [49] were employed for DNA extraction [48, 49]. Briefly, spores of pure cultures of 7-d-old *E. turcicum* grown in PDA were harvested by adding 1.5 ml 0.1% TWEEN®80 to the culture. Then, 1.2 ml of the suspension was transferred into a sterile 1.5 ml Eppendorf tube in a laminar flow hood. The suspension was centrifuged at 8,000× g for 5 min. Afterwards, the supernatant was carefully removed without disturbing the precipitate. Lysis Buffer (450 µl: 270 mM Tris, 90 mM EDTA, 1% SDS, pH 8.0) was added to each tube and vortexed briefly to resuspend the precipitate. The tubes were placed in an Eppendorf ThermoMixer® C at 60 °C and 8,000 × g for 60 min, and thereafter, centrifuged at 14,000 × g for 30 min. Then, 340 µl of the supernatant were transferred into a new, labelled sterile tube and 340 µl of refrigerated 4 M ammonium acetate were added. The suspension was thoroughly mixed. Afterwards, 680 µl ice-cold absolute ethanol was added, the content mixed, and the tube placed in a freezer at -20 °C for 30 min. The mixture was then centrifuged at 14,000 × g for 5 min and the supernatant was carefully removed. The tube was left to dry after draining the ethanol and the pellet re-suspended in 50 µl sterile nuclease-free water and gently mixed. The DNA concentration was determined using a Nanodrop spectrophotometer (Thermo Scientific™) and recorded.

### PCR primer design

Three *E. turcicum* specific primers that target effector genes (*Ecp6*, *SIX13-*like, and *SIX5-*like) were designed for the present study. Available sequences of these regions were collected from NCBI repositories, aligned and primers designed using Geneious Prime (v2020.2.4). A reference elongation factor primer (EF1-688 F/EF1-125R; [50]) was also used. The details of the four primer pairs are presented in Table 5.

**Table 5:** List of primers with sequences for *Exserohilum turcicum* effector genes

Reason for primer^a^	Primer identifier	Annealing Temp (°C)	Start	Length	Tm	Fragment size (bp)	GC%	Sequence		Remarks
Target	*SIX13*-like gene	50	78	20	59.0	169	55	TGCTAGGTGGGTCTCAGTTG	F	Moderates pathogenicity in *E. turcicum*
	*SIX13*-like gene		246	20	58.9		55	GAGAGCAGGGTCATAGGCAT	R	
Target	*SIX5*-like gene	49	219	20	59.1	215	50	ACTCGCACTCACGTAGACAA	F	Induces virulence in hemibiotrophs
	*SIX5*-like gene		433	20	58.8		55	GCGCCATAGGACTTGCATAG	R	
Target	*Ecp6*	51	143	20	59.9	316	55	TTCGGTGAACTTCCCTGTGG	F	Moderates pathogenicity in *E. turcicum*
	*Ecp6*		193	20	60.0		55	AGTGTACCAGGTGGCATTGG	R	
Reference	EF1-688 F	55	682	22	58.9	700	55	CGGTCACTTGATCTACAAGTGC	F R	(Alves et al., 2008)
	EF1-125R		1251	20	59.5		55	CCTCGAACTCACCAGTACCG		

^a^ Reason for primer: *SIX*13-like and *Ecp6* are genes that moderate pathogenicity in *E. turcicum* during the biotrophic stage, the *SIX*5-like gene induces pathogenicity in hemibiotrophs, while *-α* primer pair targets the elongation factor gene and is a reference primer

### PCR conditions and gel electrophoresis

The target fragment of each region was amplified using the following reagent concentrations: 6.0 µl OneTaq Quick-Load 2 × Master Mix, 0.24 µl 10 mM of each primer, 2 µl 20 ng/µl DNA, and 3.52 µl nuclease-free water to make a final volume of 12 µl for PCR. Initial denaturation was at 95 °C for 2 min, followed by 35 cycles of 95 °C for 30 s, annealing at 50 °C for 3 min, and extension at 72 °C for 2 min, with a final extension step at 72 °C for 25 min. The amplified fragments were separated on 1% agarose gel, which contained 1 × Tris-acetate-EDTA (TAE) buffer. The 1% agarose gel was prepared by dissolving 1 g agarose in 100 ml TAE. The solution was melted in the microwave for 5 min and allowed to cool. On cooling, 5 µl of loading dye (SafeView™ classic) was added to the solution before pouring into the electrophoresis tray and afterwards placed in a tank containing 1 × TAE for electrophoresis for 40 min at 110 V. The gel was visualized for the amplified band patterns using a gel documentation system.

## Data Availability

The datasets used in the present study have been deposited in the IITA CKANN Data Repository: 10.25502/kkcw-v956/d.

## Declarations

The laboratory experiments that were conducted complied with all relevant institutional, national, and international guidelines and legislations.

## Abbreviations

NCLB: Northern Corn Leaf Blight
SSA: sub-Saharan Africa
CWDEs: cell wall-degrading enzymes
HPR: host plant resistance
PCR: polymerase chain reaction
SIX: secreted in xylem
RMG: radial mycelia growth
PDA: potato dextrose agar
SPMF: spores per microscopic field
EDTA: ethylenediaminetetraacetic acid
SDS: sodium dodecyl sulfate
SAS: Statistical Analysis Software
SE: standard error
IITA: International Institute of Tropical Agriculture
